# A new method of subtotal thyroidectomy for Graves’ disease leaving a unilateral remnant based on the upper pole

**DOI:** 10.1097/MD.0000000000005919

**Published:** 2017-02-10

**Authors:** Yu Liu, Bin Liu, Rui-Lei Liu, Hua Jiang, Ze-Nan Huang, Yong Huang

**Affiliations:** a Department of Thyroid and Breast Surgery; b Institute of Orthopedics, The Third Affiliated Hospital of Sun Yat-Sen University, Guangzhou, China.

**Keywords:** Graves’ disease, hyperthyroidism, subtotal thyroidectomy, thyroid surgery, thyroidectomy

## Abstract

**Background:**

The aim of this prospective randomized study was to evaluate the feasibility of subtotal thyroidectomy with leaving a unilateral remnant based on the upper pole.

**Methods:**

Patients who underwent the subtotal thyroidectomy and isthmusectomy leaving either a unilateral remnant based on the upper pole (Group I, 79 patients) or the bilateral dorsal thyroid tissue remained (Group II, 89 patients) were compared in operation time, blood loss, recurrence, and postoperative complications.

**Results:**

Among 168 patients analyzed, the operation time remained similar, but the blood loss, the reoperation time, and recurrence in Group I were much less than Group II. In addition, no postoperative hemorrhage occurred in Group I. Two patients (2.28%) in Group II underwent recurrent laryngeal nerve damages. Four patients (5.06%) in Group I and 3 patients (3.37%) in Group II experienced transient hypocalcemia. Recurrence only occurred in Group II.

**Conclusion:**

In terms of blood loss, reoperation time, postoperative complication, and the recurrence, subtotal thyroidectomy with recurrent laryngeal nerves identification and the unilateral superior pole remnant of the gland provides a better outcome than subtotal thyroidectomy with bilateral dorsal thyroid tissue remnant.

## 1 Introduction

Graves’ disease (GD), an autoimmune thyroid disease, has been identified as the most common cause of hyperthyroidism. Currently, 3 different treatments are commonly adopted for GD hyperthyroidism: antithyroid drugs (ATD), radioactive iodine therapy (RAI), and surgical treatment.^[1]^ The treatment protocols for Graves’ disease diversify across countries and institutions. In most cases, ATD is often the first-line therapy modality for Graves’ disease, followed by RAI or surgery when drug therapy fails. However, surgery still has several advantages, especially in patients with a large goiter, when antithyroid medication fails, and in patients who expect an immediate remission. In these cases, the surgical procedure is still a preferred treatment of Graves’ disease.

When surgery is indicated for the treatment of GD, 1 factor that remains controversial is the extent of surgery. Total thyroidectomy (TT) leaves almost no remnant thyroid tissue behind, but it can be associated with a higher complication rate. In addition, total thyroidectomy requires patients’ lifelong self-administration of levothyroxine sodium. The majority of GD patients, particularly in developing countries such as China, are unwilling to accept this responsibility because of the long-term inconvenient life style and financial concerns. Subtotal thyroidectomy (STT) has been recommended as a safe procedure due to its lower complication rate, and thus another popular surgical treatment option.^[2–4]^ In China, a frequently adopted thyroidectomy is bilateral subtotal thyroidectomy and isthmusectomy with the posterior aspect of thyroid tissue left on either side of the trachea. However, there are several flaws in this operation. First, it is difficult to estimate the appropriate amount of remnant thyroid tissue, which may be associated with high recurrence rate of GD. Additionally, this operation mode may cause massive bleeding due to a large wound and transient or permanent recurrent laryngeal nerves damage caused by poor identification and preservation. Finally, when the illness relapses, removal of the remnant tissues may be very risky because the identification and preservation of recurrent laryngeal nerves and parathyroid gland was even more challenging.

Considering above risks, we modified the traditionally used subtotal thyroidectomy and isthmusectomy treating GD by identifying recurrent laryngeal nerves and retaining the unilateral superior pole of the gland. This study analyzed the safety and efficacy of this modified surgical procedure of Graves’ disease, thus to determine if this modified subtotal thyroidectomy could be considered a viable treatment option for patients with Graves’ disease.

## 2 Materials and methods

We confirm that the use of human subject was specifically approved by the Clinical Research Ethics Committee of the Third Affiliated Hospital, Sun Yat-sen University. Before surgery, volunteers had been informed with the possible treatment and complications, and provided their written informed consent to participate in this study. The consent procedure was approved by the Clinical Ethics Committee.

### 2.1 Study design and study population

Patients who underwent thyroidectomy from 2009 to 2014 for Graves’ disease at the Third Affiliated Hospital of Sun-Yat Sen University, Guangzhou, China, were enrolled in our study. The data were accumulated in January 2015, and all author had access to identifying information during data collection.

Patients with the following features were excluded: patients with a large thyroid tumor (diameter of single goiter ≥10 cm), patients whose thyroid remnant was less than 2 × 1 × 1 cm, and patients followed-up for less than 24 months. Patients with large thyroid tumor (diameter of single goiter ≥10 cm) were excluded because large nodule always compress remnant gland, it is hard for surgeons to save appropriate remnant gland in compressed upper pole. Patients with small remnant gland were excluded because it is hard to determine the ratio of removed gland.

Finally, a total of 168 patients with hyperthyroidism were enrolled into this study. We conducted a prospective, randomized 2-armed study, when the patients met the study criteria, they were randomized by sealed envelope into 1 of 2 surgery procedures: the subtotal thyroidectomy and isthmusectomy patients with the unilateral superior pole remained were grouped in Group I; patients who underwent the subtotal thyroidectomy and isthmusectomy with bilateral dorsal thyroid tissue remained were in Group II. The indications for surgery were persistent or recurrent hyperthyroidism after medical treatment in 128 patients (76.19%), mechanical symptoms due to a large goiter in 30 (17.86%), increased endocrine ophthalmopathy in 10 (5.95%). Antithyroid drugs were maintained or initiated in the preoperative period to bring the thyroxine and triiodothyronine levels to nontoxic values, if possible. Beta-adrenergic antagonists (β-blockers) such as propranolol or atenolol were used preoperatively in all patients to the control the adrenergic effects of excessive levels of thyroid hormones. All patients were placed on the Lugol solution for 7 to 10 days in the immediate preoperative period. Patients were ready for operation once an euthyroid state was achieved. The operational procedures in both groups were performed by 3 senior endocrine surgeons.

### 2.2 Surgery procedures

**Figure 1:**
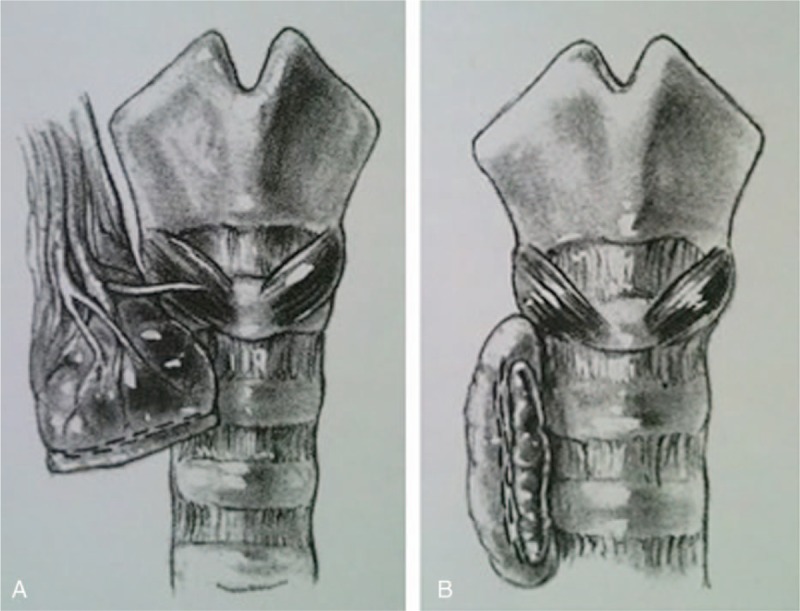
(A) Subtotal thyroidectomy and isthmusectomy with the unilateral (right) superior poles remained. (B) Subtotal thyroidectomy and isthmusectomy with bilateral dorsal thyroid tissue remained.

In patients of Group I, the enlarged glands were exposed as the routine. The middle thyroid vein was ligated, the inferior poles of the gland were lifted, and the branches of the inferior thyroid artery and veins were ligated on the capsule of the thyroid gland, superior to the origins of the blood supply to the parathyroid glands. Inferior parathyroid glands were identified and protected. At the level of the inferior thyroid artery, the bilateral recurrent laryngeal nerve was exposed, ascending slightly lateral to the tracheoesophageal groove and entering the larynx. The superior parathyroid gland was identified from the posterior aspect of the gland and pushed aside, then the majority of (larger than 80%) thyroid tissues were removed, and the upper pole on the side of the thyroid gland with relatively less disease was remained. Finally, the blood vessels were ligated (Fig. 1A).

In patients of Group II, the upper pole was freed completely and the lobe was divided along the line of resection as outlined (see Fig. 1B). Both parathyroid glands and the recurrent nerve were presumed to be left in their normal locations, not being exposed as routine. The gland between hemostats was divided until the anterior surface of the trachea was reached. The lateral margin of the residual segment of thyroid was sutured to the trachea.

### 2.3 Follow-up protocol

Follow-up data were obtained at the routine clinic visit. All patients were followed up by clinical examination, laryngoscopy, ultrasound, and blood test every 3 months for the first 2 years. We defined recurrent laryngeal nerve damage as vocal cord paralysis confirmed by laryngoscopy, hypocalcaemia as serum-ionized calcium less than 1.1 mmol/L, postop hypothyroidism as the elevated TSH value. Information about all these complications was gleaned.

### 2.4 Statistical analysis

Postoperative variables reviewed were blood loss, mean operative time, recurrent laryngeal nerve damage, hypocalcemia (transient or permanent), recurrence, and reoperative time. Quantitative variables were expressed as mean and compared using Student's *t*-test. As appropriate, quantitative variables were expressed as numbers with percentages and compared with *x*^2^ or Fisher's exact test. Statistical analysis for Student's *t*-test and *x*^2^ analysis were performed using SPSS16.0. A *P*-value <0.05 was considered statistically significant.

## 3 Results

In the study period, 168 patients (117 females and 51 males) received surgery. Their age ranged from 25 to 65 years, with a median age of 43.7 years. However, 79 cases were randomly categorized in Group I, whereas 89 were in Group II. The 2 groups were not significantly different in gender, age, and surgical indications (see Table 1). Mean observation periods in Group I is 34.9 ± 9.7 months and in Group II, it is 35.1 ± 8.3 (*P* = 0.918, 95%CI = −2.9 to 2.6).

**Table 1:**
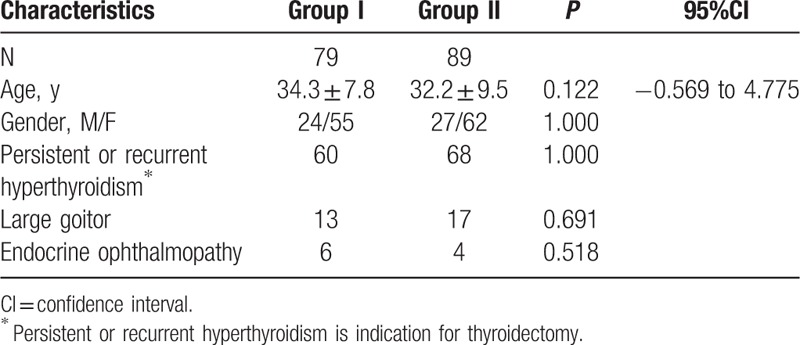
The patients’ general information.

**Figure 2:**
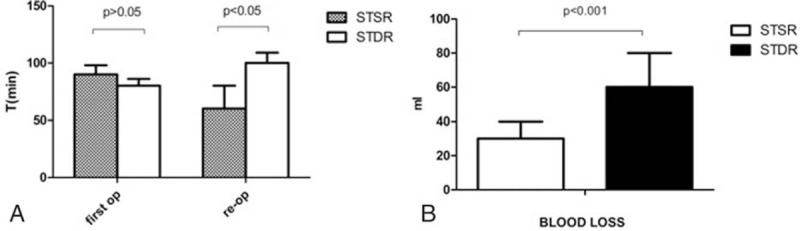
(A) The first operation time and reoperation time between group I and group II. There is no significant difference between the 2 groups (*P* > 0.05). The reoperation time in group I was much less than that in group II (*P* < 0.05). (B) The blood loss between group I and group II. The mean estimated blood loss in group I (30 ± 10 mL) was much less than that in group II (60 ± 20 mL) (*P* < 0.001).

No significant difference (*P*-value > 0.05) in the average operative time was observed in 2 groups, with 90 ± 8 minutes in Group I, and 85 ± 13 minutes in Group II (Fig. 2). However, the mean estimated blood loss (EBL) in Group I (30 ± 10 mL) was much less than that in Group II (60 ± 20 mL) (*P*-value < 0.05), and the GD recurrence rate was much higher in Group II (3.80% vs 7.87%, *P*-value < 0.05) (see Fig. 2B). When Graves’ disease relapses, the first-line treatment is usually antithyroid medication, but reoperation is a secondary treatment when patients have serious medication contraindications or with suspected thyroid cancer. GD reoccurred in 3 patients in Group I and in 7 patients in Group II. Among them, 2 patients in Group I and 3 patients in Group II received reoperation. The reoperation time for Group I patients (40 ± 6 min) was less than in Group II (100 ± 9 min) (*P*-value < 0.01). The estimated blood loss is 32.5 ± 3.5 mL in Group I and 60 ± 10 mL in Group II (*P* = 0.037,95%CI = −51.9 to −3.0). In Group I, there was no recurrent laryngeal nerve damage or postoperative hypocalcemia. In Group II, there was 1 patient had recurrent laryngeal nerve damage, 3 patients had postoperative hypocalcemia (1 was transient and 2 were permanent) (see Table 2).

**Table 2:**
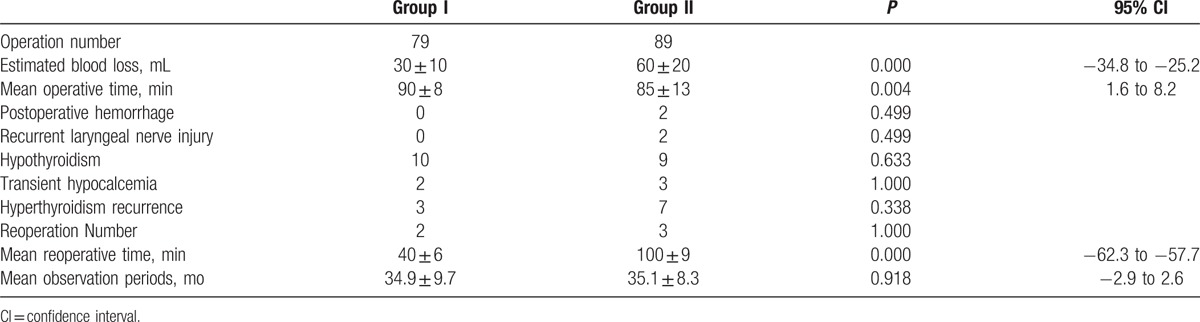
Operative variables and postoperative follow-up.

Both groups were followed up from 24 months to 60 months. No significant difference was found in the occurrence of hypothyroidism in both groups, and all of them achieved euthyroidism after treating with levothyroxine for 6 to 12 months.

Table 2 also indicates the postoperative complications. No cases of postoperative hematoma were found in Group I, whereas 2 patients in Group II (2.25%) underwent reoperations for a cervical hematoma from a bleeding strap muscle (same day). 2 patients in Group II (2.25%) suffered from recurrent laryngeal nerve damage, but recovered half a year later. 2 patients (2.53%) in Group I and 3 patients (3.37%) in Group II experienced transient hypocalcemia. Recurrence only occurred in 2 patients in Group II (2.25%). No permanent hypocalcemia was identified in both groups. In Group I, 3 patients (3.80%) developed recurrent disease. In Group II, 7 patients (7.87%) recurrence after surgery. Because the remnant gland volume is difficult to evaluate in traditional subtotal thyroidectomy, recurrence is frequent when too much gland left, this kind of recurrence is hardly controlled by ATDs, so these 10 patients urged to undergo total thyroidectomy.

## 4 Discussion

Previous studies have shown that subtotal thyroidectomy is a safe and effective treatment for Graves’ disease.^[5–7]^ However, there are still substantial debates regarding the size of resection and the option of operation modalities. Associated with a lower risk of developing recurrence of disease, total thyroidectomy has become the first-line treatment option for patients with Graves’ disease in developed countries.^[8–9]^ Although total thyroidectomy prevents relapse of the disease, it renders patients postoperative hypothyroidism, even permanently, thus often adopted in compliance with postoperative thyroid hormone supplementation, which requires patients’ responsibility for lifelong self-administration. For majority of Chinese GD patients, they are unwilling to accept this responsibility because of this long-term inconvenient life style.

The traditional subtotal thyroidectomy may have the following risks.^[10]^ First, it may cause greater estimated blood loss, particularly in patients with an enlarged goiter, mostly likely due to bleeding from the cut surface of a highly vascular and enlarged thyroid gland. Second, the bilateral recurrent laryngeal nerves are not routinely identified in this treatment, increasing the possibility of damage. Third, the blood supply to the parathyroid glands during the operation may become insufficient because the inferior thyroid artery is divided, resulting postoperative hypocalcemia. Fourth, when recurrences occur and a completion thyroidectomy is needed, the bilateral recurrent laryngeal nerves are even more easily damaged because of the adhesion resulted from the first operation.^[11–12]^ Fifth, long-term follow-up showed a 18% hyperthyroidism recurrence because of the difficult and inaccurate estimate of residual thyroid.^[13]^

**Figure 3:**
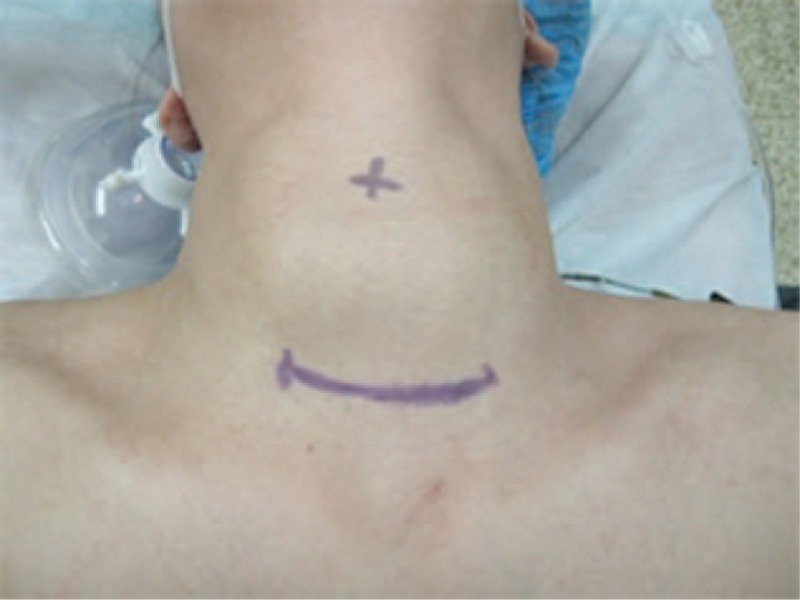
Scope of incision was marked on the neck skin.

**Figure 4:**
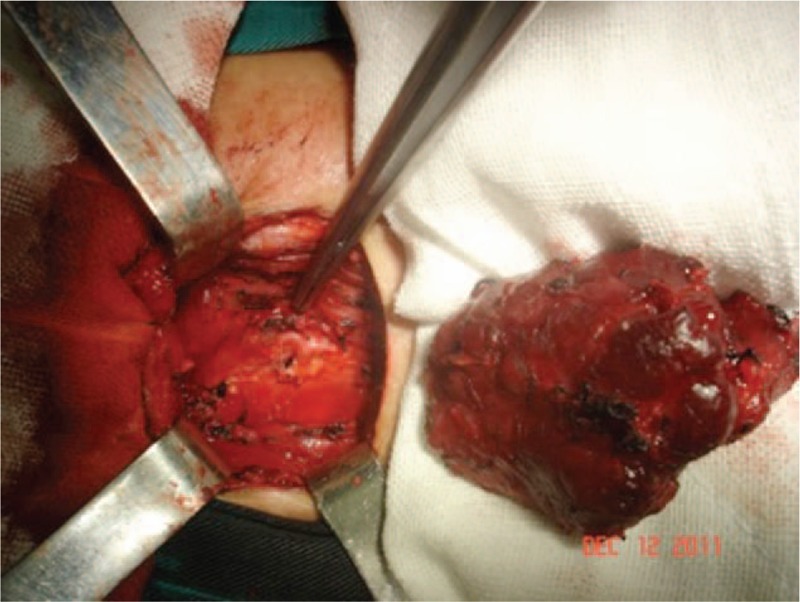
Right RLN was exposed and protected with superior parathyroid remained carefully. RLN = recurrent laryngeal nerves.

**Figure 5:**
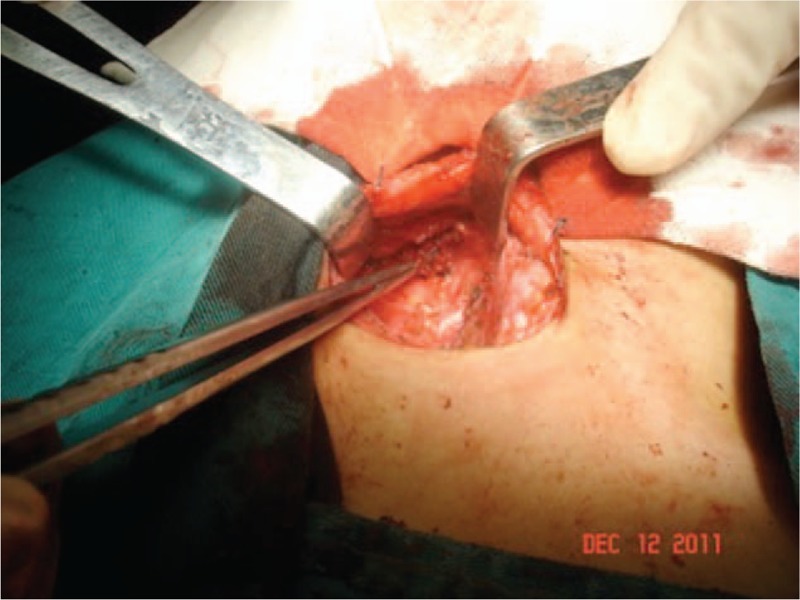
Subtotal thyroidectomy and isthmusectomy with the right superior poles remained.

In this study, a modified subtotal thyroidectomy was employed to treat Graves’ disease in Chinese patients (Figs. 3–5). In this treatment mode, the bilateral recurrent laryngeal nerve was carefully identified and preserved (see Fig. 4). Operative visualization of the recurrent laryngeal nerves throughout the entire operation was important in eliminating permanent vocal cord paralysis. The thyroid tissue was resected with approximately 3 gram (2 cm × 1cm × 1cm) of unilateral upper pole thyroid gland (5%) remained (see Fig. 5). Sufficient blood supply to the parathyroid glands was ensured by ligating the branches of the inferior thyroid artery and veins on the capsule of the thyroid gland, superior to the origins of the blood supply to the parathyroid glands, which would be important in reducing the incidence of permament hypoparathyroidism. We named this surgery mode as the subtotal thyroidectomy and isthmusectomy with the unilateral superior poles remnant.

In our modified subtotal thyroidectomy with the unilateral superior poles remnant, the bilateral recurrent laryngeal nerves (RLN) were routinely identified and preserved, and the unilateral superior lobes of the thyroid gland were reserved. This modified surgery mode has the following advantages. Preserving the superior lobes can markedly reduce blood loss during operation due to a smaller cut surface, a stronger ligation and without involving the superior thyroid artery, and less blood loss will promise a clean and clear surgery field to ensure the surgeon protect RLN and parathyroid gland effectively. It was effective to protect the bilateral recurrent laryngeal nerves by active identification before resection (0 vs 2). This result was consistent with Riddecl's finding that the rate of RLN damage could be reduced from 2% to 0.6% with regular identification. To protect the parathyroid, we kept the inferior artery trunk for sufficient blood supply and retained the superior parathyroid in case of mistakenly cutting of inferior parathyroid. Only 2 patients had transient hypocalcemia and recovered soon in Group I, indicating that the new treatment was safe for parathyroid. The recurrence of GD in Group II was obviously higher than that in Group I (7 vs 3, *P* = 0.037). Perhaps because in patients with large goiters and symptoms of mechanical compression in the middle and inferior lobes, it was easier to estimate the extent of thyroidectomy and to determine the weight of resected thyroid tissue by cutting the middle and inferior lobes and preserving the superior lobes. No patients in Group I had cervical hematoma postoperatively, whereas 2 patients in Group II had hematoma and needed reoperation, and hematoma were confirmed coming from strap muscle, probably because of the less clear surgery field due to more bleeding during the first operation.

With all the results, we conclude that subtotal thyroidectomy leaving a unilateral remnant based on the upper pole performs an effective and safe surgical way for Grave's disease and is associated with less injury and complications. Due to a lack of cases, more data from multiple centers are needed for further studies.
